# Enterovirus serotypes in patients with central nervous system and respiratory infections in Viet Nam 1997–2010

**DOI:** 10.1186/s12985-018-0980-0

**Published:** 2018-04-12

**Authors:** Nguyen Thi Thuy Chinh B’Krong, Ngo Ngoc Quang Minh, Phan Tu Qui, Tran Thi Hong Chau, Ho Dang Trung Nghia, Lien Anh Ha Do, Nguyen Ngoc Nhung, Nguyen Van Vinh Chau, Guy Thwaites, Le Van Tan, H. Rogier van Doorn, Tran Tan Thanh

**Affiliations:** 1 Oxford University Clinical Research Unit, Ho Chi Minh City, Viet Nam; 2 Division of Medicine and Laboratory Science, University of Oslo, Oslo, Norway; 3 Children’s Hospital 1, Ho Chi Minh City, Viet Nam; 4 Hospital for Tropical Diseases, Ho Chi Minh City, Viet Nam; 5 Murdoch Children’s Research Institute, Melbourne, Australia; 6 Department of Biotechnology, University of Science, Ho Chi Minh City, Viet Nam; 7 Centre for Tropical Medicine and Global Health, Nuffield Department of Medicine, University of Oxford, Oxford, UK; 8 Oxford University Clinical Research Unit, 78 Giai Phong, Dong Da, Ha Noi, Viet Nam

**Keywords:** Enterovirus, Serotyping, Central nervous system infection, Respiratory infection

## Abstract

**Background:**

Enteroviruses are the most common causative agents of human illness. Enteroviruses have been associated with regional and global epidemics, recently, including with severe disease (Enterovirus A71 and D68), and are of interest as emerging viruses. Here, we typed *Enterovirus A-D* (EV) from central nervous system (CNS) and respiratory infections in Viet Nam.

**Methods:**

Data and specimens from prospective observational clinical studies conducted between 1997 and 2010 were used. Species and serotypes were determined using type-specific RT-PCR and viral protein 1 or 4 (VP1, VP4) sequencing.

**Results:**

Samples from patients with CNS infection (51 children – 10 CSF and 41 respiratory/rectal swabs) and 28 adults (28 CSF) and respiratory infection (124 children – 124 respiratory swabs) were analysed. Twenty-six different serotypes of the four Enterovirus species (A-D) were identified, including EV-A71 and EV-D68. Enterovirus B was associated with viral meningitis in children and adults. Hand, foot and mouth disease associated Enteroviruses A (EV-A71 and Coxsackievirus [CV] A10) were detected in children with encephalitis. Diverse serotypes of all four Enterovirus species were found in respiratory samples, including 2 polio-vaccine viruses, but also 8 CV-A24 and 8 EV-D68. With the exception of EV-D68, the relevance of these viruses in respiratory infection remains unknown.

**Conclusion:**

We describe the diverse spectrum of enteroviruses from patients with CNS and respiratory infections in Viet Nam between 1997 and 2010. These data confirm the global circulation of Enterovirus genera and their associations and are important for clinical diagnostics, patient management, and outbreak response.

**Electronic supplementary material:**

The online version of this article (10.1186/s12985-018-0980-0) contains supplementary material, which is available to authorized users.

## Background

Enteroviruses are non-enveloped single stranded RNA viruses of the genus *Enterovirus* within the family of *Picornaviridae*. Seven species of *Enterovirus* are associated with human disease: *Enterovirus A-D* and *Rhinovirus A-C*. While rhinoviruses commonly cause mild respiratory illness, enteroviruses A-D (EVs) are a significant cause of morbidity and mortality worldwide. Prior to being reclassified as EV A-D, EVs were originally classified as polioviruses (PV) 1–3, coxsackieviruses (CV) A1–24 and B1–6, echoviruses (E) 1–33 and numbered enteroviruses (68–121) [1].

In addition to the surveillance system for poliomyelitis, some countries have established comprehensive surveillance programs for circulating non-polio EVs [2, 3]. In recent years, surveillance activities have been enhanced in response to the emergence of hand, foot, and mouth disease (HFMD) causing enteroviruses in the Asia-Pacific region [4] and the global spread of EV-D68 causing respiratory infections [5, 6].

EV infections are often asymptomatic, but may also result in a diverse spectrum of clinical illness, varying from mild febrile illnesses to severe disease of the cutaneous, gastrointestinal, respiratory, cardiovascular, and central nervous system (CNS) [7, 8]. Generally, EV A is associated with herpangina and hand, foot and mouth disease (HFMD), EV B with herpangina and sporadic and epidemic viral meningitis or encephalitis, EV C with poliomyelitis and EV D with respiratory infections [2, 9–11]. In Viet Nam, since 2005, various serotypes of EV A*,* most commonly enterovirus A71 (EV-A71), coxsackievirus A16 (CV-A16), CV-A10, and CV-A6 have been associated with outbreaks of HFMD [12, 13] and EVs have also been frequently detected in aetiological studies of CNS and respiratory infections [14–18].

In the majority of aetiological studies only generic RT-PCR is used for detection of enteroviruses [14–16, 18, 19]. Information about specific enterovirus serotypes circulation and their associated clinical phenotypes therefore remains sparse.

Here we report the clinical associations and serotyping results of EVs that were previously detected in our studies of CNS and respiratory infections in southern and central Viet Nam between 1997 and 2010.

## Methods

### Patients and clinical samples

Data and samples from 5 prospective observational clinical studies on CNS (*n* = 3) and respiratory (*n* = 2) infections, conducted in Viet Nam between 1997 and 2010 were used [14–16, 18, 19]. Patients were enrolled according to study-specific case definitions for CNS infection, acute respiratory infection and lower respiratory tract infection. The three CNS studies were conducted to determine the etiology of CNS infections in children in Children’s Hospital 1 in Ho Chi Minh City [HCMC] (2004), and in adults in the Hospital for Tropical Diseases (1997–2008) and in a network of 12 provincial hospitals in the southern and central part of Viet Nam (2007–2010). In these three studies, CSF was obtained from all participants, while throat and rectal swabs were only taken from children. The two respiratory infections studies were conducted to study lower respiratory tract infection in hospitalized children under 2 years of age in two main paediatric hospitals in HCMC (2009–2010) and the antibiotic use in out-patients with acute respiratory infections in Children’s Hospital 1 in HCMC (2009–2010). Respiratory swabs were taken from all enrolled children. Patients positive for generic EV RT-PCRs in CSF or swabs from these studies and with diagnostic specimens available were included in this study. The geographical distribution of included patients is shown in Additional file 1: Figure S1.

### Nucleic acid extraction

Viral RNA was extracted from clinical samples using either the MagNA Pure 96 platform (Roche Applied Science, Darmstadt, Germany) or the QIAamp Viral RNA Mini kit (QIAgen GmbH, Hilden, Germany) following the manufacturer’s instructions.

### EV-A71 specific one-step real-time RT-PCR

EV-A71 detection and typing of samples from patients with CNS infection was performed using real-time RT-PCR as described previously [12]. In brief, 2 μl of viral RNA were subjected to one-step real-time RT-PCR reaction using the SuperScript III One-Step qRT-PCR system with Platinum Taq DNA Polymerase (Invitrogen, Carlsbad, CA, USA). The cycling conditions included one cycle of 60 °C for 3 min, followed by 15 min at 53 °C and 2 min at 95 °C, and 45 cycles of 15 s at 95 °C, 1 min at 53 °C (including data acquisition) and 15 s at 72 °C.

### Nested RT-PCRs and sequencing

Nested RT-PCR for viral protein 1 (VP1) and complete viral protein 4 and partial viral protein 2 (VP4/VP2) was carried out using previously described assays [20–22]. VP1 RT-PCR of samples from patients with CNS infection was primarily performed using the assay described by Leitch et al., [22] and alternatively, the VP1 assay described by Oberste et al., [20]. The VP4/VP2 assay described by Mirand et al., [21] was applied to all samples from patients with respiratory infection and to samples from patients with CNS infection with negative VP1 RT-PCRs. There was no major modification of the original assays except the use of Superscript III reverse transcriptase with Platinum *Taq* (Invitrogen) in the first round RT-PCR and Hotstar Taq DNA polymerase (QIAgen) in the second round PCR and the adaptation of thermal cycling conditions as per supplier’s instructions. In the three assays, 5 μl of viral RNA were used in the first round one-step RT-PCR and then 0.5 μl of the first round PCR product was transferred to the second round PCR reaction.

PCR products of the 2nd round PCR were analysed in agarose gel, purified with cold ethanol and were then subjected to DNA sequencing using the Big Dye Terminator v3.1 Cycle Sequencing Kit (Applied Biosystems Inc., Foster City, CA, USA).

### Nucleotide sequence and phylogenetic tree analyses

Nucleotide sequences of the VP1 and VP4/VP2 were assembled using Vector NTI ® Express software v7.1 (Thermo Fisher Scientific, Waltham, USA). All the sequences have been submitted to GenBank (MH021887-MH021955). Multiple sequence alignments were performed using BioEdit software v7.0.9 (Ibis Therapeutics, CA, USA) and with the inclusion of reference prototype sequences obtained from GenBank. Neighbour-joining trees were constructed in MEGA software v7.0.26 (www.megasoftware.net) using the maximum composite likelihood nucleotide substitution model with 1000 bootstrap replicates.

### Serotype determination

Serotype assignments for VP1 and VP4/VP2 sequences were performed using an automated phylogenetic-based enterovirus typing tool developed by Kroneman et al., [23] available at http://www.rivm.nl/mpf/enterovirus/typingtool/. Because the automated typing tool can determine VP4/VP2 sequences at species level only [23], further serotype determination for VP4/VP2 sequences was based on highest nucleotide identity score (HNIS) and highest amino acid sequence similarity (HAASS) with reference prototypes [24, 25]. In case of discrepancy, final assignment was based on HAASS and further confirmed using BLAST [21, 26].

### Data analysis

Statistical analyses were performed using SPSS v23.0 (SPSS, Inc., Chicago, IL, USA). Categorical variables were compared using Chi-square or Fisher’s exact tests and continuous variables were compared using Mann-Whitney U test. Two-sided *P* values ≤0.05 were considered significant.

## Results

### Patient characteristics

**Fig. 1:**
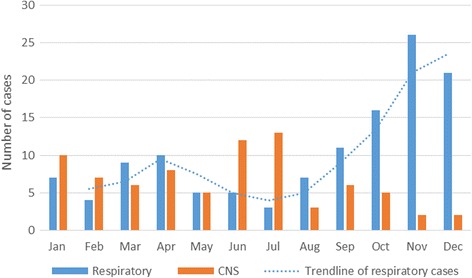
Monthly distribution of cases with EV detection

Samples from a total of 203 patients were included in this study, including from 79 patients with CNS infection and 124 with respiratory infection. When analyzing for the monthly distribution of cases, there was no clear peak among CNS cases, whereas two peaks of respiratory cases were found in April and November (Fig. 1).

Among patients with CNS infection (*n* = 79), 51 were children (median age 2 years, interquartile range [IRQ]: 1–5) and 28 were adults (20 years, IRQ: 17–29.5). All adults had EV detected in CSF and 93% (26/28) had meningitis as discharge diagnosis. Of the 51 children, 10 had EV detected in CSF, and the remaining (41/51) had EV detected in respiratory and/or rectal swabs. Encephalitis was the most common discharge diagnosis (30/41). Fifteen deaths were noted, and all were children with EV detected in swabs only (Table 1).

**Table 1:** Demographic and clinical data from 79 patients with CNS infection and enterovirus detection

		Children ≤15 years (*n* = 51)		Adults > 15 years (*n* = 28)	*P* value^c^
		Swab (*n* = 41)	CSF (*n* = 10)	CSF (*n* = 28)	
Demographics	Male (%)	29 (70.7)	3 (30)	18 (64.3)	0.078
	Age (year) (median; IQR)	1 (1–3)	7 (1.8–10)	20 (17–29.5)	ND
Discharge diagnosis	Encephalitis (%)	30 (73.2)	4 (40)	1 (3.6)	0.012
	Meningitis (%)	2 (4.9)	6 (60)	26 (92.9)	
	Others^b^(%)	9 (22)	0	1 (3.6)	
Clinical symptoms	Fever	40 (97.6)	9 (90)	26 (92.9)	1.0
	Day of illness (med: IQR)	4 (3–5)	2 (1.5–3.5)	2 (1–3)	0.699
	Duration of hospitalization (med: IQR)	7 (2.5–12)	7 (6.5–10.5)	13 (5–15)	0.173
	Meningeal signs (%)	4 (9.8)	4 (40)	20 (71.4)	0.127
	Convulsions (%)	26 (63.4)	2 (20)	0	0.064
	Focal neurology (%)	22 (53.7)	1 (10)	0	0.263
	Limb weakness (%)	22 (53.7)	1 (10)	0	0.263
	GCS < 9 (%)	25 (61)	1 (10)	1 (3.6)	0.462
Laboratory tests	Blood white cell count (per mm3) [med: IQR]	12,300	16,800	9750	0.010
		(8150–16,225)	(14875–22,800)	(7100–11,950)	
	Blood neutrophil % (med: IQR)	53.15 (36–69.8)	80.8 (73–86.4)	78.35 (73.1–82.6)	0.713
	Blood glucose (mmol per litter) [med: IQR]	5.16 (4.0–7.8)	5.22 (3.8–6.5)	6.04 (5.17–7.1)	0.311
	CSF white cell counts per mm3 (med: IQR)	5 (1–45.5)	61 (3.5–172)	90.5 (27.25–197.5)	0.926
	CSF neutrophil % (med: IQR)	20 (11–30)	63 (37.5–67.5)	46 (27.8–78.8)	0.596
	CSF glucose (mmol per litter) [med: IQR]	3.5 (2.8–4.7)	3.74 (3.4–4.3)	3.5 (3.16–3.9)	0.380
	CSF protein (med: IQR)	0.2 (0.1–0.3)	0.42 (0.28–0.49)	0.5 (0.39–0. 7)	0.362
	Lactate (mmol per litter) [med: IQR]	2.09 (1.5–3)	2.35 (1.9–7.5)	2.45 (2.1–3.1)	1.00
	Evidence of other pathogens^a^ in CSF (%)	13 (31.7)	1 (10)	3 (10.7)	1.0
Co-infection^a^	JEV (n)	9	1	0	NA
	DENV (n)	4	0	0	
	*Cytomegalovirus* (n) [CMV]	0	0	2	
	*Mycobacterium tuberculosis* (n) [MTB]	0	0	1	
Outcome	Death (%)	15 (36.6)	0	0	NA

*ND* not determined, *NA* not applicable

Note: ^a^Co-infection included detection of human IgM (anti JEV and DENV) and viral DNA (CMV) in CSF, or bacterial growth (*Mycobacterium tuberculosis*) in CSF culture

^b^Other discharge diagnoses included sepsis (*n* = 5), dengue haemorrhagic fever (*n* = 1), colitis (*n* = 1), diarrhea (*n* = 1) metabolic disease (*n* = 1), and hepatitis (*n* = 1)

^c^Statistical comparison between children and adults with EV detection in CSF

Among patients with respiratory infections, the median age was 12.7 months (IQR: 5.7–24.3). Inpatients (*n* = 65) were younger than outpatients (*n* = 59), due to study enrolment criteria (Table 2). Bronchiolitis (as assessed at the discretion of treating physicians) was the most common clinical diagnosis (57% in outpatients and 61% in inpatients). Thirty-five percent of inpatients had a clinical diagnosis of pneumonia (Table 2).

**Table 2:** Demographic and clinical data from 124 children with respiratory infection and enterovirus detection

		All patient	In-patient	Out-patient (*n* = 59)	*P* value
		(*n* = 124)	(*n* = 65)		
Demographics	Male (%)	83 (66.9)	45 (69.2)	38 (64.4)	0.703
	Age (month) (median; IQR)	12.65 (5.7–24.3)	6.07 (3.1–11.7)	25.34 (15.5–38.2)	< 0.001
Clinical diagnosis	Bronchiolitis (%)	73 (58.9)	37 (56.9)	36 (61)	0.716
	Pneumonia (%)	23 (18.5)	23 (35.4)	0	< 0.001
Clinical symptoms	Fever (%)	38 (30.6)	29 (44.6)	9 (15.3)	< 0.001
	Runny nose (%)	104 (83.9)	55 (84.6)	49 (83.1)	1
	Cough (%)	123 (99.2)	64 (98.5)	59 (100)	1
Co-infection^a^	Rhinovirus (%)	85 (68.5)	63(96.9)	22 (37.3)	< 0.001
	Respiratory syncytial virus (%)	34 (27.4)	27 (41.5)	7 (11.9)	< 0.001
	Adenovirus (%)	17 (13.7)	5 (7.7)	12 (20.3)	0.065
	Parainfluenza (%)	11 (8.9)	6 (9.2)	5 (8.5)	1
	Human metapeumovirus (%)	11 (8.9)	3 (4.6)	8 (13.6)	0.114
	Human bocavirus (%)	8 (6.5)	6 (9.2)	2 (3.4)	0.278
	Human coronavirus [HCoV] (%)	5 (4)	3 (4.6)	2 (3.4)	1
	Influenza virus (%)	3 (2.4)	0	3 (5.1)	0.105
	Parechovirus [PEV] (%)	1 (0.8)	1 (1.5)	0	1
Outcome	Death (%)	0	0	0	NA

Note: ^a^indicates confirmed by specific (RT)PCR; *NA* not applicable, *IQR* interquartile range

### Co-infection with other pathogens

In patients with CNS infection, co-infection was more common and was found in 27% (14/51) children versus 11% (3/28) adults (Table 1). Most co-infected children (13/14) had EV detected in swabs only. Of the 15 documented fatal cases, 7 were serologically confirmed to have co-infection with dengue virus (DENV; *n* = 3) and Japanese encephalitis virus (JEV; *n* = 4) (Additional file 4: Table S1). Among the 3 co-infected adults, 2 had cytomegalovirus (CMV) DNA detected in CSF and 1 had positive CSF culture with *Mycobacterium tuberculosis* (Table 1).

In both patient groups of respiratory infection, there were high rates of co-infection with other respiratory viruses. Co-infection with rhinovirus (RHV; 69%) and respiratory syncytial virus (RSV; 27%) was more common than other viruses, including adenovirus (ADV; 14%), parainfluenza viruses (PIV; 9%), human metapneumovirus (hMPV; 9%), human bocavirus (BoV; 7%), human coronavirus (HCoV; 4%), influenza virus (Flu; 2%), and parechovirus (PEV; 1%). The rate of co-infection with RHV and RSV was significantly higher in the inpatient group as compared to the outpatient group (*P* < 0.001) (Table 2).

### Enterovirus serotypes in CNS and respiratory infections

Enterovirus serotype determination was successful in 38% (77/203) of patients: 19/38 (50%) CSFs (6 children, 13 adults) and 27/41 (66%) swabs from patients with CNS infections, and 31/124 (25%) of swabs from patients with respiratory infections. A total of 26 different serotypes belonging to four enterovirus species (A-D) were identified.

Twenty-one different serotypes belonging to 3 enterovirus species: EV A (*n* = 12, 3 serotypes), B (*n* = 30, 16 serotypes), and C (*n* = 4, 2 serotypes) were identified in patients with CNS infection. The identification of these serotypes was achieved by EV-A71 specific RT-PCR (*n* = 8; Additional file 4: Table S2), VP1 (*n* = 22; Additional file 2: Figure S2) and VP4/VP2 (*n* = 16; Additional file 3: Figure S3) sequencing. EV B was the only species detected in adults and the most common species in both swabs and CSF from children (Table 3). EV-A71 was the most commonly detected single serotype, in 9 swabs and 1 CSF of 10 children. Three of these children had a clinical diagnosis of HFMD while the other 7 children had a clinical diagnosis of encephalitis (Additional file 4: Table S2). There was no specific serotype associated with fatal outcome (Additional file 4: Table S1).

**Table 3:** Enterovirus species and serotypes in clinical samples from patients with CNS or respiratory infection

Enterovirus species	CNS cases			Respiratory cases		
	Serotype	Children (*n* = 33)	Adults (*n* = 13)	Serotype	Inpatients (*n* = 15)	Outpatients (*n* = 16)
A	CV-A10	1	.	CV-A6	1	.
	CV-A12	1	.	EV-A90	1	.
	EV-A71*	10	.	.	.	.
B	CV-A9	.	1	CV-A9	.	2
	CV-B1	1	.	CV-B1	.	2
	CV-B2	1	.	CV-B4	.	1
	CV-B4	1	.	CV-B5	.	2
	CV-B5	2	1	E-7	.	2
	E-4	1	4	E-12		1
	E-6	3	.	E-30	1	.
	E-9	.	2	.	.	.
	E-12	2	.	.	.	.
	E-16	.	1	.	.	.
	E-18	1	.	.	.	.
	E-19	2	.	.	.	.
	E-24	1	.	.	.	.
	E-25	1	.	.	.	.
	E-27	.	1	.	.	.
	E-30	1	3	.	.	.
C	EV-C96	3	.	CV-A24	3	5
	PV-2	1	.	PV-1	1	.
	.	.	.	PV2	1	.
D	.	.	.	EV-D68	7	1

Note: * indicates identified by EV-A71 specific RT-PCR (*n* = 8) and VP4/VP2 sequencing (*n* = 2)

Among patients with respiratory disease 13 different serotypes of four enterovirus species were identified by VP4/VP2 sequence analysis including A (*n* = 2, 2 serotypes), B (*n* = 11, 7 serotypes), C (*n* = 10, 3 serotypes), and D (*n* = 8, 1 serotype). CV-A24 (*n* = 8) and EV-D68 (*n* = 8) were the two most common serotypes detected (Table 3) and these viruses had a close genetic relationship in phylogenetic tree analyses (Additional file 3: Figure S3).

## Discussion

There is limited information on circulating enterovirus serotypes and associated clinical phenotypes from the Asia Pacific region, including Viet Nam. Such knowledge is essential for laboratory diagnostics, patient management and future outbreak response.

Here, the diversity of enterovirus serotypes belonging EV A-D was assessed in samples from patients with CNS and respiratory infections. Some of these serotypes, including EV-A71 and EV-D68 have recently been recognized as emerging viral pathogens with pandemic potential.

EV-A71 is the most important pathogen of HFMD epidemics in the Asia-Pacific region with over 2 million cases reported annually [27, 28]. Here, EV-A71 was detected in 10/51 (9 swabs and 1 CSF) children with CNS infections, three of those had a clinical diagnosis of HFMD of which two died [13]. In 9/10 cases, EV-A71 was the only pathogen detected in diagnostic samples. These data are in accordance with previous reports showing that EV-A71 has the potential to cause brain stem encephalitis and death in children under 5 years of age [27], but is rarely detected in CSF [29].

EV-D68 has recently been recognized as an important cause of respiratory illness [30]. EV-D68 emergence was first reported from two children’s hospitals in the USA in August 2014 [31] and, shortly after, from countries across the Americas, Europe, and Asia [30]. Here, EV-D68 accounted for 8/31 successfully typed enteroviruses from patients with respiratory illness recruited between 2009 and 2010. It remains unknown why EV-D68 has been circulating in Viet Nam for some time but has not been associated with outbreaks as observed in the USA and other countries in Southeast Asia. We have recently reported an in depth phylogenetic analysis of these EV-D68 viruses showing that their introduction into Viet Nam may have already occurred in 2008 [32].

CV-A24 has been associated with herpangina, acute flaccid paralysis and epidemics of acute haemorrhagic conjunctivitis [33]. In the present study, it was one of the most common enteroviruses: 8/31 typed viruses from patients with respiratory illness. However, the circulation of this particular enterovirus serotype has not been reported from Viet Nam.

EV B was found in CSF of both children and adults and 3/4 children and 11/13 adults had a clinical diagnosis of meningitis (Additional file 4: Table S4), in accordance with the known clinical spectrum of EV B [9, 10]. EV A was found in 2 CSF of children and both had a clinical syndrome of encephalitis (Additional file 4: Table S4). It is likely these children had HFMD associated rhombencephalitis, in line with reports from the region starting in 1997 [27]. Remarkably, none of the 8 children who died had either EV or any other pathogen detected in CSF suggesting other etiology and/or emphasizing the diagnostic challenges of encephalitis [14, 34].

In addition to EV-A71, other common serotypes in CNS infections cases included E-4 and E-30 (both EV B). These two viruses have frequently been involved in outbreaks of aseptic meningitis worldwide [35, 36]. While E-30 has been confirmed to be the cause of a meningoencephalitis outbreak in northern Viet Nam previously [37], E-4 has yet been associated with any local epidemic of viral meningitis among children and young adults.

PV-1 and 2 (EV C) were detected in swab specimens of 3 children: one with CNS and two with respiratory illness. As children were under 2 and sequences had 99–100% of VP4/VP2 identity to Sabin vaccine strains [38] these were considered to be derived from oral polio vaccine.

Data from this study showed that among patients with CNS infection, children had a higher rate of co-infection than adults. Here, this was due to the fact that both swabs and CSF were used in the diagnostics for children while only CSF was used for adults. In addition, children may be more vulnerable to viral infection than adults, particularly to the locally endemic viruses JEV (*n* = 10) and DENV (*n* = 4) [14]. As JEV and DENV infections are commonly associated with severe disease and mortality, the contribution of EV to the disease phenotype of these cases is uncertain. Similarly, in patients with respiratory infection, the clinical significance of EV infection should be interpreted with caution because of the high rate of co-infection with more common respiratory viruses [39].

All the serotyping assays used in the current study, including the EV-A71 specific RT-PCR, VP1 and VP4/VP2 sequencing have previously shown their efficient performance in clinical samples. The application of these assays in samples of patients with CNS infection would help to improve serotyping yield. However, the overall serotyping success rate for patients with CNS infection in the current study was low (58%) as compared to previous studies [21, 22]. This may be due to low viral load or nucleic acid degradation after long-term storage. The low serotyping success rate (25%) of respiratory samples can be explained by the sole use of VP4/VP2 sequencing assay. However, sample degradation and false positivity for EV using generic PCR among RV co-infected patients (68.5%) could not be excluded. Other drawbacks of the current study included the small sample size, the sporadic and expanded geographical distribution of cases that hampered our further analyses for serotype-associated seasonality and geographics [7]. Despite these limitations, however, our study revealed diverse enterovirus serotypes have been circulating in Viet Nam in association with respiratory and CNS infections.

## Conclusions

Our study illustrates the circulation of diverse enterovirus serotypes belonging to four species (A-D), and their association with respiratory and CNS infections in Viet Nam. These data are important for patient management, laboratory diagnostics and future outbreak response.

## Additional files

Additional file 1: **Figure S1.** The geographic distribution of CNS and respiratory cases included in the current study (TIFF 892 kb)

Additional file 2: **Figure S2.** Phylogenetic tree analysis of partial VP1 enteroviral sequences. A middle-point rooted tree of partial VP1 sequences (330 bp) showing the genetic relationship among species and serotypes of the current study (filled triangles) and with reference prototypes. Filled triangles indicate sequences derived from patients with CNS infection. (TIFF 5689 kb)

Additional file 3: **Figure S3.** Phylogenetic tree analysis of VP4/VP2 enteroviral sequences. A middle-point rooted tree of VP4/VP2 (420 bp) sequences showing the genetic relationship among species and serotypes of the current study (filled triangles and circles) and with reference prototypes. Filled triangles indicate sequences derived from patients with CNS infection and filled circles indicate sequences derived from patients with respiratory infection. (TIFF 1075 kb)

Additional file 4: **Table S1.** Demographic, clinical and diagnostic information of the 15 death cases. Table S2. Demographic, clinical and laboratory information of 10 cases with EV-A71 infection. Table S3. VP4/VP2 nucleotide and amino acid similarity scores. Table S4. Demographic, clinical and laboratory of 19 cases with CSF enterovirus serotypes. (DOCX 34 kb)
