# Supplementary figures and images for: Enterovirus serotypes in patients with central nervous system and respiratory infections in Viet Nam 1997–2010
Source: Virol J. 2018 Apr 12;15:69. doi: 10.1186/s12985-018-0980-0 (PMC5897964; doi:10.1186/s12985-018-0980-0)

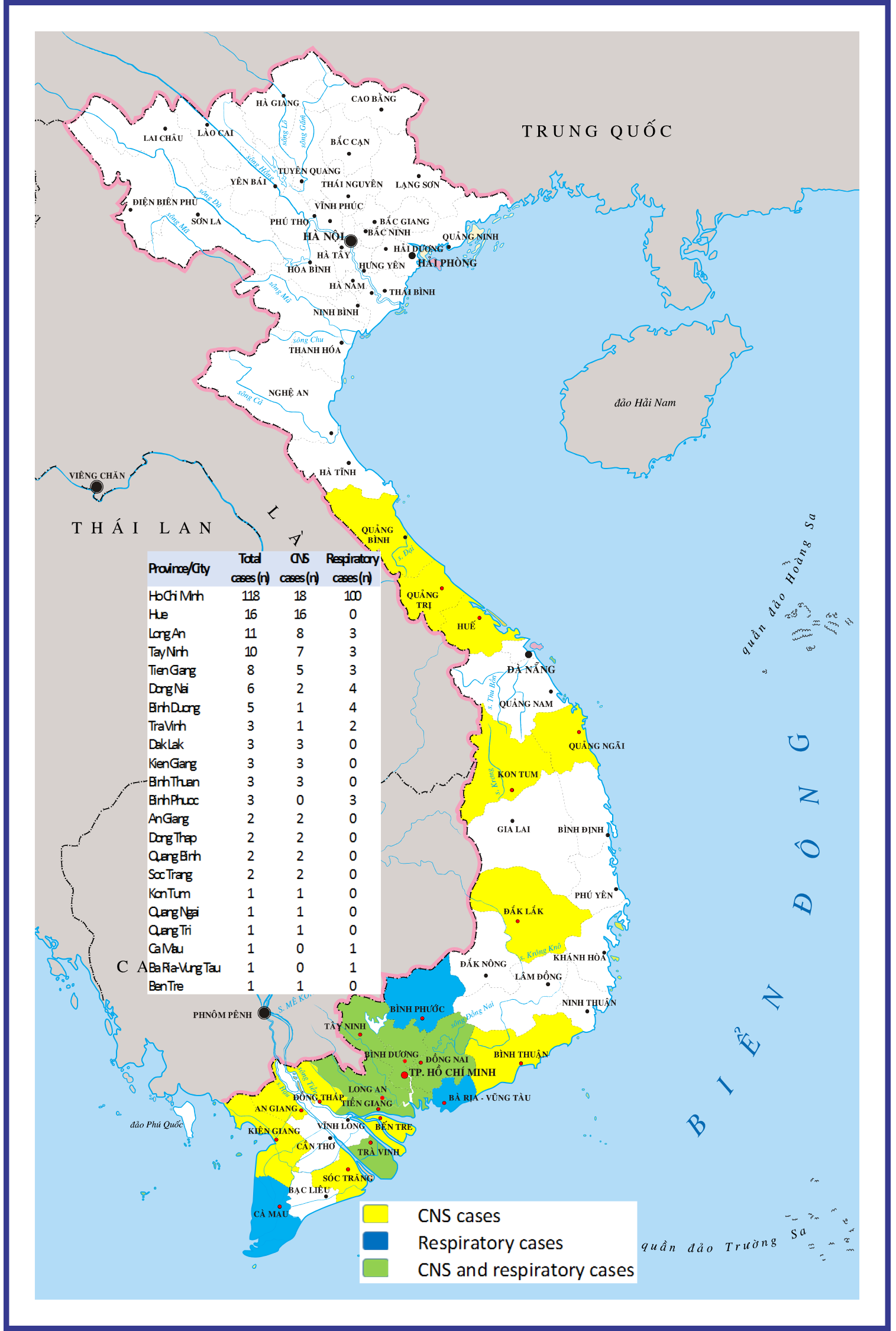

Supplement: Supplementary file 1 — Figure S1. The geographic distribution of CNS and respiratory cases included in the current study (TIFF 892 kb) [file 12985_2018_980_MOESM1_ESM.tif]

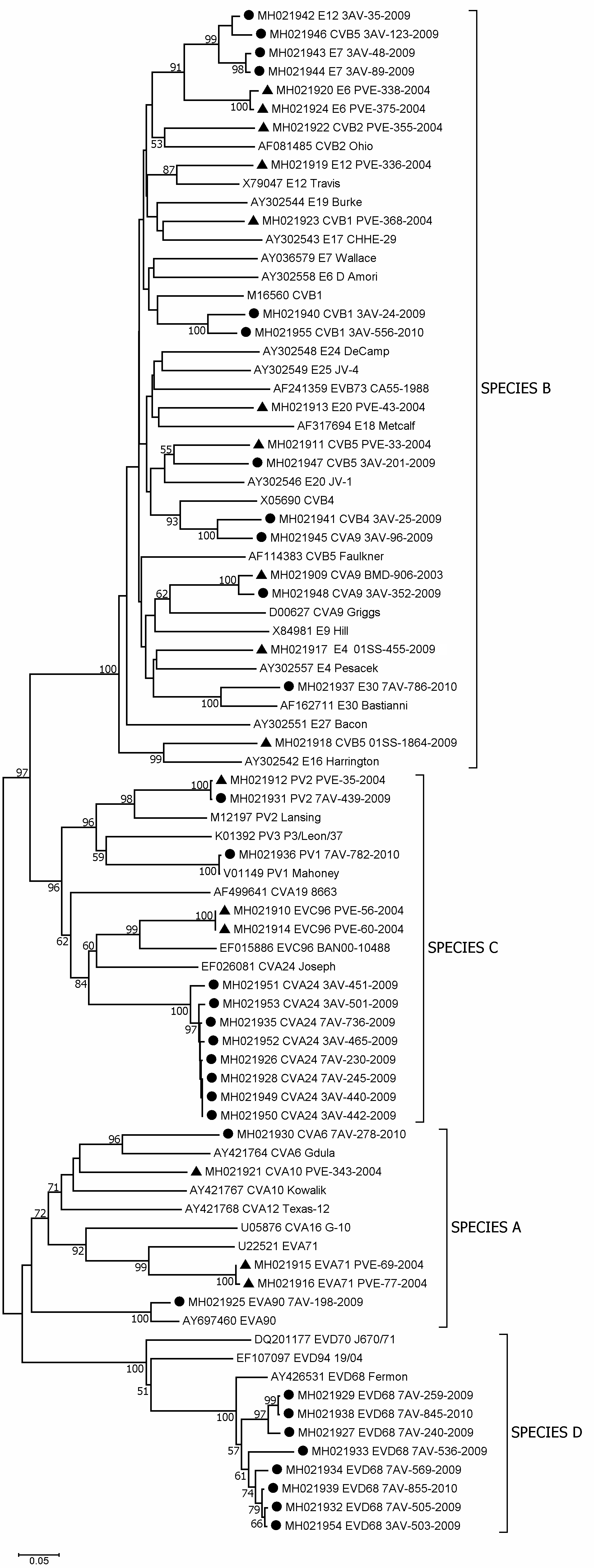

Supplement: Supplementary file 3 — Figure S3. Phylogenetic tree analysis of VP4/VP2 enteroviral sequences. A middle-point rooted tree of VP4/VP2 (420 bp) sequences showing the genetic relationship among species and serotypes of the current study (filled triangles and circles) and with reference prototypes. Filled triangles indicate sequences derived from patients with CNS infection and filled circles indicate sequences derived from patients with respiratory infection. (TIFF 1075 kb) [file 12985_2018_980_MOESM3_ESM.tiff]
