# Supplementary material for: Enterovirus serotypes in patients with central nervous system and respiratory infections in Viet Nam 1997–2010
Source: Virol J. 2018 Apr 12;15:69. doi: 10.1186/s12985-018-0980-0 (PMC5897964; doi:10.1186/s12985-018-0980-0)
Supplement: Supplementary file 4 — Table S1. Demographic, clinical and diagnostic information of the 15 death cases. Table S2. Demographic, clinical and laboratory information of 10 cases with EV-A71 infection. Table S3. VP4/VP2 nucleotide and amino acid similarity scores. Table S4. Demographic, clinical and laboratory of 19 cases with CSF enterovirus serotypes. (DOCX 34 kb) [file 12985_2018_980_MOESM4_ESM.docx]

Table S1: Demographic, clinical and diagnostic information of the 15 death cases

| Death cases (No) | Year of enrolment | Age (year) | Sex | Illness day at admission | Duration of hospitalization (day) | Discharge diagnosis | Glasgow coma score | Sample with enterovirus | Enterovirus serotypes | *Evidence of other pathogens in CSF |
| --- | --- | --- | --- | --- | --- | --- | --- | --- | --- | --- |
| 1 | 2004 | 0 | F | 5 | 41 | Sepsis | 8 | TS | E-25 |  |
| 2 | 2004 | 11 | F | 4 | 9 | Encephalitis | 3 | TS and RS | EV-A71 | DENV |
| 3 | 2004 | 2 | M | 4 | 19 | Metabolic disease | 9 | TS and RS | CV-B5 |  |
| 4 | 2004 | 0 | M | 3 | 7 | Sepsis | 10 | RS | CV-B5 |  |
| 5 | 2004 | 5 | F | 3 | 18 | Sepsis/hepatitis | 6 | RS | EV-96 |  |
| 6 | 2004 | 0 | M | 2 | 6 | Encephalitis | 12 | TS | NA | DENV |
| 7 | 2004 | 0 | M | 5 | 11 | Dengue hemorrhagic fever | 13 | RS | EV-96 | DENV |
| 8 | 2004 | 3 | F | 4 | 8 | Encephalitis | 14 | RS | NA | JEV |
| 9 | 2004 | 3 | M | 4 | 8 | Encephalitis | 14 | TS and RS | EV-A71 |  |
| 10 | 2004 | 2 | M |  | 5 | Febrile convulsions | 13 | RS | E-6 | JEV |
| 11 | 2004 | 1 | M | 5 | 5 | Encephalitis | 10 | TS | NA |  |
| 12 | 2004 | 1 | M | 2 | 3 | Encephalitis | 15 | TS and RS | CV-B1 |  |
| 13 | 2004 | 6 | F | 3 | 7 | Encephalitis | 4 | TS | NA | JEV |
| 14 | 2004 | 2 | F | 2 | 7 | Encephalitis | 12 | RS | E-24 |  |
| 15 | 2004 | 4 | M | 6 | 6 | Encephalitis | 9 | TS | NA | JEV |

Note: *Evidenced by IgM assays or specific PCRs. M = male; F = female; TS = throat swab; RS = rectal swab; DENV = dengue virus; JEV = Japanese encephalitis virus

Table S2: Demographic, clinical and laboratory information of 10 cases with EV-A71 infection

| Patient (No) | Year of enrolment | Age (year) | Sex | Illness day at admission | Duration of hospitalization (day) | Discharge diagnosis | Glasgow coma score | Samples | Typing method | *Other pathogens in CSF | Outcome |
| --- | --- | --- | --- | --- | --- | --- | --- | --- | --- | --- | --- |
| 1 | 2004 | 11 | F | 4 | 9 | encephalitis | 3 | TS and RS | RT-PCR | DENV | D |
| 2 | 2004 | 1 | M | 4 | 1 | HFMD/  encephalitis | 3 | TS and RS | RT-PCR | . | S |
| 3 | 2004 | 2 | M | 5 | 0 | encephalitis | 6 | TS and RS | RT-PCR | . | S |
| 4 | 2004 | 1 | F | 5 | 1 | encephalitis | 3 | RS | VP4/VP2 | . | S |
| 5 | 2004 | 1 | F | 3 | 0 | encephalitis | 3 | TS and RS | PCR | . | S |
| 6 | 2004 | 0 | M | 4 | 1 | encephalitis | 3 | TS and RS | VP4/VP2 | . | S |
| 7 | 2004 | 3 | M | 4 | 8 | HFMD/  encephalitis | 14 | TS and RS | RT-PCR | . | D |
| 8 | 2004 | 2 | F | 3 | 27 | HFMD/  encephalitis | 13 | CSF | RT-PCR | . | S |
| 9 | 2004 | 1 | M | 5 | 8 | encephalitis | 11 | RS | RT-PCR | . | S |
| 10 | 2004 | 1 | M | 3 | 1 | encephalitis | 5 | RS | RT-PCR | . | S |

Note: *indicates detected by IgM assay. M = male; F = female; TS = throat swab; R = rectal swab; D = died; S = survived.

Table S3: VP4/VP2 nucleotide and amino acid similarity scores

| **GenBank Accession** | **Isolate** | **Species** | **VP4/VP2 nucleotide score** | | **VP4/VP2 amino acid score** | | **Serotype designation** | **Note** |
| --- | --- | --- | --- | --- | --- | --- | --- | --- |
|  |  |  | Highest identity (%) | Type | Highest identity (%) | Type |  |  |
| MH021915 | PVE-69-2004 | A | **81.43** | **EV-A71** | **99.20** | **EV-A71** | **EV-A71** | **CNS cases** |
| MH021916 | PVE-77-2004 | A | **80.90** | **EV-A71** | **97.60** | **EV-A71** | **EV-A71** |  |
| MH021921 | PVE-343-2004 | A | **76.39** | **CV-A8** | **96. 80** | **CV-A10** | **CV-A10** |  |
| MH021917 | 1SS-455-2009 | B | **79.04** | **E-4** | **95.05** | **E-4** | **E-4** |  |
| MH021918 | 1SS-1864-2009 | B | **80.24** | **E-16** | **97.02** | **E-16** | **E-16** |  |
| MH021909 | BMD-906-2003 | B | **81.14** | **EV-75** | **98.02** | **CV-A9** | **CV-A9** |  |
| MH021911 | PVE-33-2004 | B | *82.34* | *E-20* | 97.03 | CV-B5 | **CV-B5** |  |
| MH021913 | PVE-43-2004 | B | **80.24** | **E-33** | **97.03** | **CV-B4** | **CV-B4** |  |
| MH021919 | PVE-336-2004 | B | **83.53** | **E-12** | **99.01** | **E-12** | **E-12** |  |
| MH021920 | PVE-338-2004 | B | 80.54 | E-6 | **96.04** | **E-6** | **E-6** |  |
| MH021922 | PVE-355-2004 | B | **79.64** | **CV-B3** | **97.03** | **CV-B2** | **CV-B2** |  |
| MH021923 | PVE-368-2004 | B | **82.04** | **E-12** | **97.03** | **CV-B1** | **CV-B1** |  |
| MH021924 | PVE-375-2004 | B | **81.44** | **E-6** | **97.03** | **E-6** | **E-6** |  |
| MH021912 | PVE-35-2004 | C | **83.56** | **PV-2** | **97.74** | **PV-2** | **PV-2** |  |
| MH021910 | PVE-56-2004 | C | **83.71** | **EV-C96** | **99.25** | **EV-C96** | **EV-C96** |  |
| MH021914 | PVE-60-2004 | C | **83.71** | **EV-C96** | **99.25** | **EV-C96** | **EV-C96** |  |
| MH021930 | 7AV-278-2010 | A | **80.51** | **CV-A6** | **92.43** | **CV-A6** | **CV-A6** | **Respiratory cases** |
| MH021925 | 7AV-198-2009 | A | **94.62** | **EV-A90** | **98.57** | **EV-A90** | **EV-A90** |  |
| MH021940 | 3AV-24-2009 | B | **82.09** | **CV-B1** | **97.58** | **CV-B1** | **CV-B1** |  |
| MH021941 | 3AV-25-2009 | B | *82.67* | *CV-B4* | 98.40 | CV-B4 | **CV-B4** |  |
| MH021942 | 3AV-35-2009 | B | *81.72* | *CV-B1* | 93.55 | E-12 | **E-12** |  |
| MH021943 | 3AV-48-2009 | B | **81.07** | **E-19** | **94.40** | **E-7** | **E-7** |  |
| MH021944 | 3AV-89-2009 | B | **80.80** | **E-19** | **95.20** | **E-7** | **E-7** |  |
| MH021945 | 3AV-96-2009 | B | 83.73 | CV-B4 | **96.00** | **CV-A9** | **CV-A9** |  |
| MH021946 | 3AV-123-2009 | B | **82.74** | **E-17** | **96.38** | **CV-B5** | **CV-B5** |  |
| MH021947 | 3AV-201-2009 | B | **81.87** | **CV-B5** | **97.60** | **CV-B5** | **CV-B5** |  |
| MH021948 | 3AV-352-2009 | B | **81.07** | **CV-A9** | **97.60** | **CV-A9** | **CV-A9** |  |
| MH021955 | 3AV-556-2010 | B | **82.13** | **E-19** | **98.40** | **CV-B1** | **CV-B1** |  |
| MH021937 | 7AV-786-2010 | B | **86.76** | **E-30** | **94.07** | **E-30** | **E-30** |  |
| MH021950 | 3AV-442-2009 | C | **76.90** | **EV-C102** | **96.18** | **EV-A24** | **CV-A24** |  |
| MH021949 | 3AV-440-2009 | C | **76.90** | **EV-C102** | **96.18** | **EV-A24** | **CV-A24** |  |
| MH021951 | 3AV-451-2009 | C | **77.66** | **EV-C96** | **96.18** | **EV-A24** | **CV-A24** |  |
| MH021952 | 3AV-465-2009 | C | **76.12** | **EV-C102** | **95.42** | **EV-A24** | **CV-A24** |  |
| MH021953 | 3AV-501-2009 | C | **76.90** | **CV-A13** | **95.42** | **EV-A24** | **CV-A24** |  |
| MH021926 | 7AV-230-2009 | C | **76.90** | **CV-A13** | **96.18** | **EV-A24** | **CV-A24** |  |
| MH021928 | 7AV-245-2009 | C | **76.90** | **EV-C102** | **96.18** | **EV-A24** | **CV-A24** |  |
| MH021931 | 7AV-439-2009 | C | **83.76** | **PV-2** | **97.71** | **PV-2** | **PV-2** |  |
| MH021935 | 7AV-736-2009 | C | **76.90** | **EV-C102** | **96.18** | **EV-A24** | **CV-A24** |  |
| MH021936 | 7AV-782-2010 | C | **99.75** | **PV-1** | **99.24** | **PV-1** | **PV-1** |  |
| MH021954 | 3AV-503-2009 | D | **91.56** | **EV-D68** | **100** | **EV-D68** | **EV-D68** |  |
| MH021927 | 7AV-240-2009 | D | **89.61** | **EV-D68** | **100** | **EV-D68** | **EV-D68** |  |
| MH021929 | 7AV-259-2009 | D | **90.58** | **EV-D68** | **100** | **EV-D68** | **EV-D68** |  |
| MH021932 | 7AV-505-2009 | D | **91.88** | **EV-D68** | **100** | **EV-D68** | **EV-D68** |  |
| MH021933 | 7AV-536-2010 | D | **90.26** | **EV-D68** | **96.08** | **EV-D68** | **EV-D68** |  |
| MH021934 | 7AV-569-2010 | D | **91.56** | **EV-D68** | **100** | **EV-D68** | **EV-D68** |  |
| MH021938 | 7AV-845-2010 | D | **90.58** | **EV-D68** | **100** | **EV-D68** | **EV-D68** |  |
| MH021939 | 7AV-855-2010 | D | **91.56** | **EV-D68** | **100** | **EV-D68** | **EV-D68** |  |

Table S4: Demographic, clinical and laboratory of 19 cases with CSF enterovirus serotypes

| Patient (No) | Year of enrolment | Age (year) | Sex | Illness day at admission | Duration of hospitalization (day) | Discharge diagnosis | Glasgow coma score | Enterovirus | | *Other pathogens in CSF | Outcome |
| --- | --- | --- | --- | --- | --- | --- | --- | --- | --- | --- | --- |
|  |  |  |  |  |  |  |  | Species | Serotypes |  |  |
| 1 | 2004 | 2 | F | 3 | 27 | Encephalitis | 13 | A | EV-A71 |  | S |
| 2 | 2004 | 13 | M | 4 | 7 | Encephalitis | 10 | A | CV-A10 | JEV | S |
| 3 | 2010 | 6 | F | 1 | 10 | Meningitis | 15 | B | E-30 |  | S |
| 4 | 2009 | 8 | F | 2 | 7 | Encephalitis | 15 | B | E-4 |  | S |
| 5 | 2008 | 2 | F |  | 5 | Meningitis | 15 | B | E-18 |  | S |
| 6 | 2010 | 8 | M | 1 | 7 | Meningitis | 15 | B | E-19 |  | S |
| 7 | 1998 | 28 | M | 5 | 7 | Meningitis | 15 | B | CV-B5 |  | S |
| 8 | 1998 | 19 | M | 2 | 4 | Meningitis | 15 | B | E-27 |  | S |
| 9 | 2000 | 34 | M | 3 | 3 | Meningitis | 15 | B | E-9 |  | S |
| 10 | 2000 | 18 | M | 3 | 4 | Meningitis | 15 | B | E-9 |  | S |
| 11 | 2003 | 35 | M | 2 | 3 | Meningitis | 15 | B | CV-A9 | CMV | S |
| 12 | 2007 | 20 | F | 2 | 4 | Encephalitis | 15 | B | E-30 |  | S |
| 13 | 2007 | 19 | M | 2 | 5 | Meningitis | 15 | B | E-30 |  | S |
| 14 | 2009 | 22 | F | 4 | 5 | Meningitis | 15 | B | E-4 |  | S |
| 15 | 2009 | 17 | F | 1 | 15 | Meningitis | 15 | B | E-16 |  | S |
| 16 | 2009 | 35 | M | 3 | 11 | Meningitis | 15 | B | E-4 |  | S |
| 17 | 2009 | 16 | M | 2 | 14 | Meningitis | 15 | B | E-4 |  | S |
| 18 | 2009 | 20 | M | 1 | 14 | Meningitis | 15 | B | E-30 |  | S |
| 19 | 2009 | 83 | M | 1 | 16 | Colitis | 15 | B | E-4 |  | S |

Note: *Other pathogens detected by IgM assay (JEV) or specific PCR (CMV). M = male; F = female; S = survive.
